# Transitional correlation between inner-membrane potential and ATP levels of neuronal mitochondria

**DOI:** 10.1038/s41598-018-21109-2

**Published:** 2018-02-14

**Authors:** R. Suzuki, K. Hotta, K. Oka

**Affiliations:** 1 Centre for Biosciences and Informatics, School of Fundamental Sciences and Technology, Keio University, 3-14-1 Hiyoshi, Kohoku-ku, Yokohama, Kanagawa 223-8522 Japan; 2 Graduate Institute of Medicine, College of Medicine, Kaohsiung Medical University, Kaohsiung, Taiwan

## Abstract

The importance of highly active mitochondria and their contribution to neuronal function has been of recent interest. In most cases, however, mitochondrial activity is estimated using measurements of mitochondrial inner membrane potential (IMP_mito_), and little is known about the dynamics of native mitochondrial ATP (ATP_mito_). This study conducted simultaneous imaging of IMP_mito_ and ATP_mito_ in neurons to explore their behaviour and their correlation during physiological mitochondrial/neuronal activity. We found that mitochondrial size, transport velocity and transport direction are not dependent on ATP_mito_ or IMP_mito_. However, changes in ATP_mito_ and IMP_mito_ during mitochondrial fission/fusion were found; IMP_mito_ depolarized *via* mitochondrial fission and hyperpolarized *via* fusion, and ATP_mito_ levels increased after fusion. Because the density of mitochondria is higher in growth cones (GCs) than in axonal processes, integrated ATP_mito_ signals (density × ATP_mito_) were higher in GCs. This integrated signal in GCs correlated with axonal elongation. However, while the averaged IMP_mito_ was relatively hyperpolarized in GCs, there was no correlation between IMP_mito_ in GCs and axonal elongation. A detailed time-course analysis performed to clarify the reason for these discrepancies showed that IMP_mito_ and ATP_mito_ levels did not always correlate accurately; rather, there were several correlation patterns that changed over time.

## Introduction

Mitochondria are organelles that produce adenosine triphosphate (ATP), a major energy source for living bodies, and are known to be enriched in regions that have a high energy demand at both the organ and subcellular levels^1–3^. This is especially true for neurons due to their highly polarised morphology, which is indispensable for proper neuronal activities, and local activity that requires in-place and highly effective energy production *via* mitochondria. In fact, neuronal mitochondria are enriched in specific cellular regions with large energy demands, such as synapses, nodes of Ranvier, and growth cones (GCs)^4,5^. Although action potential generation is known to be a major ATP consumer in neurons^4,6,7^, morphological changes and neurite growth also require substantial amounts of energy, especially during developmental stages^8–11^.

Although mitochondria have a variety of roles, ATP production is considered a major function, because the inhibition of oxidative phosphorylation results in stalled mitochondria and defective neuronal behaviour^8,12–15^. Therefore, ATP and mitochondria are frequently considered to be closely related to cellular viability or health. For example, many neurodegenerative diseases with a loss of neuronal function and morphology have accompanying mitochondrial dysfunctions^16–18^. Additionally, in some neuronal injury models, unimpaired mitochondria are crucial for neuronal energy recovery and neuronal survive^18,19^. In this context, the existence of healthy mitochondria seems to be interpreted as sufficient ATP availability in neurons, although in most cases mitochondrial ATP (ATP_mito_) was not measured. There are some studies showed mitochondrial activity; however, mitochondrial inner membrane potential (IMP_mito_) was frequently investigated as surrogate measure of ATP_mito_, despite no direct demonstration of correlation in physiological conditions between them.

Furthermore, previous findings regarding neuronal mitochondria were derived from experiments using artificial manipulation of mitochondrial functions, such as knockdown of mitochondria-specific proteins required for docking to motor proteins or for fission/fusion^8,20,21^. However, mitochondria in neurites are regulated by multiple, complex mechanisms and several properties exhibit large variety, such as transport (direction, velocity, proportion of moving mitochondria, and anchoring or docking), distribution, fission and fusion, and morphology (size or aspect ratio), other than functional aspects such as IMP_mito_ and ATP_mito_^5,11^. Moreover, mutual relationships between some of these properties have recently been revealed. For example, knockdown of the fusion-related protein, OPA1, resulted in changes to mitochondrial size and morphology, as well as transport velocity and distribution^20^. The dominant negative form of TRAK2, a kinesin adaptor protein, also caused defective mitochondrial transport, as well as reduced IMP_mito_^9^. Furthermore, a consensus regarding a mutual relationship between IMP_mito_ and direction of transport has not been established. Miller and Sheetz suggested that 90% of mitochondria with high IMP_mito_ move anterogradely, whereas 80% of those with low IMP_mito_ move retrogradely^22^, while another study showed no correlation between IMP_mito_ and the direction of axonal transport^23^. Due to the mutual dependency and complexity of several mitochondrial properties, it is necessary to study mitochondrial function without artificial interruption to clarify native mitochondrial behaviour.

To clarify native mitochondrial behaviour, especially regarding energy metabolism abilities, direct measurements of ATP_mito_, IMP_mito_ and other properties such as transport, morphology or distribution under physiological condition are necessary. Although mitochondrial isolation^24^ is one of effective method for characterisation of a mitochondrion, some property such as transport or distribution need to be measured within cells. However, little research has been conducted investigating ATP_mito_ measurements under physiological conditions or with other mitochondrial parameters in living cells. There are several reasons for this: (1) there are limited methods to directly measure ATP_mito_ with high spatiotemporal resolution^25,26^; (2) in general, change in ATP levels without extensive stimulation is subtle, and therefore hard to evaluate; and (3) assessing a variety of properties simultaneously requires elaborate and careful preparation, conditioning and treatment in both experimentation and analysis. However, we recently used spatiotemporal image processing analysis to overcome such problems and have demonstrated relationships between biological signals collected from simultaneous fluorescent imaging under physiological conditions^27,28^.

With this background, we conducted simultaneous fluorescent imaging of ATP_mito_, IMP_mito_, and other mitochondrial or neuronal properties in neurons using mitAT1.03^25,29,30^, an ATP_mito_ indicator, and tetramethylrhodamine ethyl ester (TMRE)^31,32^, an IMP_mito_ indicator, in this present study. Detailed analysis of the relationships revealed that not all ATP_mito_ and IMP_mito_ correlated accurately, and as for axonal elongation, ATP_mito_ is more a dominant factor than IMP_mito_.

## Results

### IMP_mito_ and ATP_mito_ in transport

**Figure 1:**
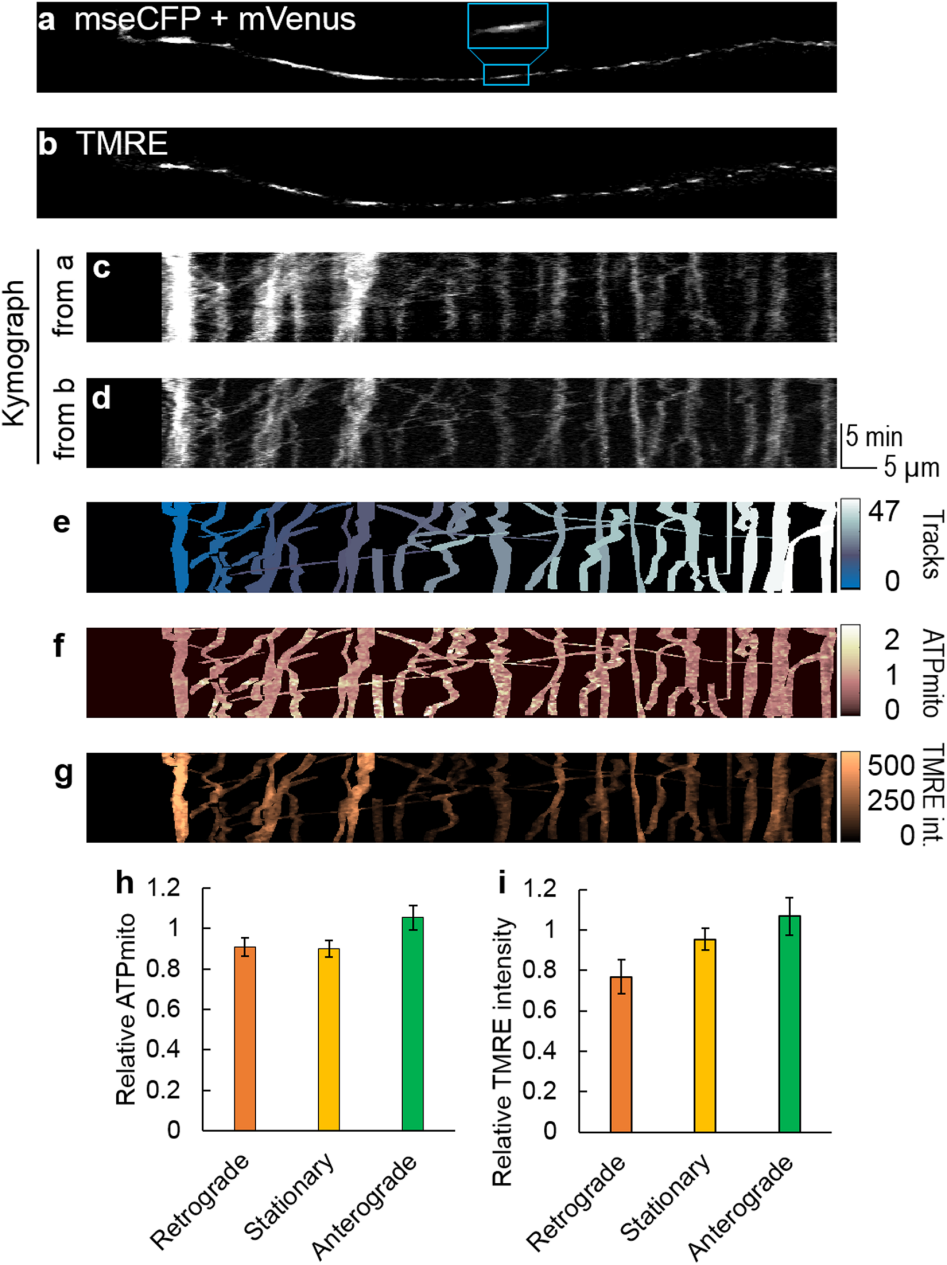
Mitochondrial ATP and inner-membrane potential dependency on the transport direction of mitochondria. A typical image of a nerite visualised for (**a**) mseCFP + mVenus fluorescence (mitAT1.03) or (**b**) TMRE. Acquired kymograph from a (**c**) and b (**d**). (**e**) Manually identified mitochondrial tracks. Here, the growth cone area was excluded from kymograph. (**f**) Mitochondrial ATP or (**g**) TMRE intensity visualised in pseudo colour. Each colour table is linear and covers the full range of the data. Relative (**h**) mitochondrial ATP and (**i**) TMRE intensity levels for 33 retrograde, 78 stationary and 27 anterograde mitochondria from 50 axonal processes. Error bars represent the standard error of the mean (SEM).

Firstly, to investigate ATP_mito_ or IMP_mito_ dependency on transportation, we compared ATP_mito_ or IMP_mito_ among anterogradely transported, stationary and retrogradely transported mitochondria within axonal processes using kymographs (Figs 1, S1, S2). Mitochondria that moved anterogradely had relatively higher levels of ATP_mito_; however, the difference was not significant (Fig. 1h). IMP_mito_ was relatively depolarized in retrogradely transported mitochondria compared to anterogradely transported mitochondria. However, there was no significant difference between the IMP_mito_ of anterogradely transported mitochondria and stationary mitochondria (Fig. 1i). No ATP_mito_ or IMP_mito_ dependence on mitochondrial velocity and transported distance was found (Supplementary Fig. S3). Results of ATP_mito_ and IMP_mito_ were expected to be similar; however, our result showed that they did not accurately coincide.

### IMP_mito_ and ATP_mito_ in fission/fusion

**Figure 2:**
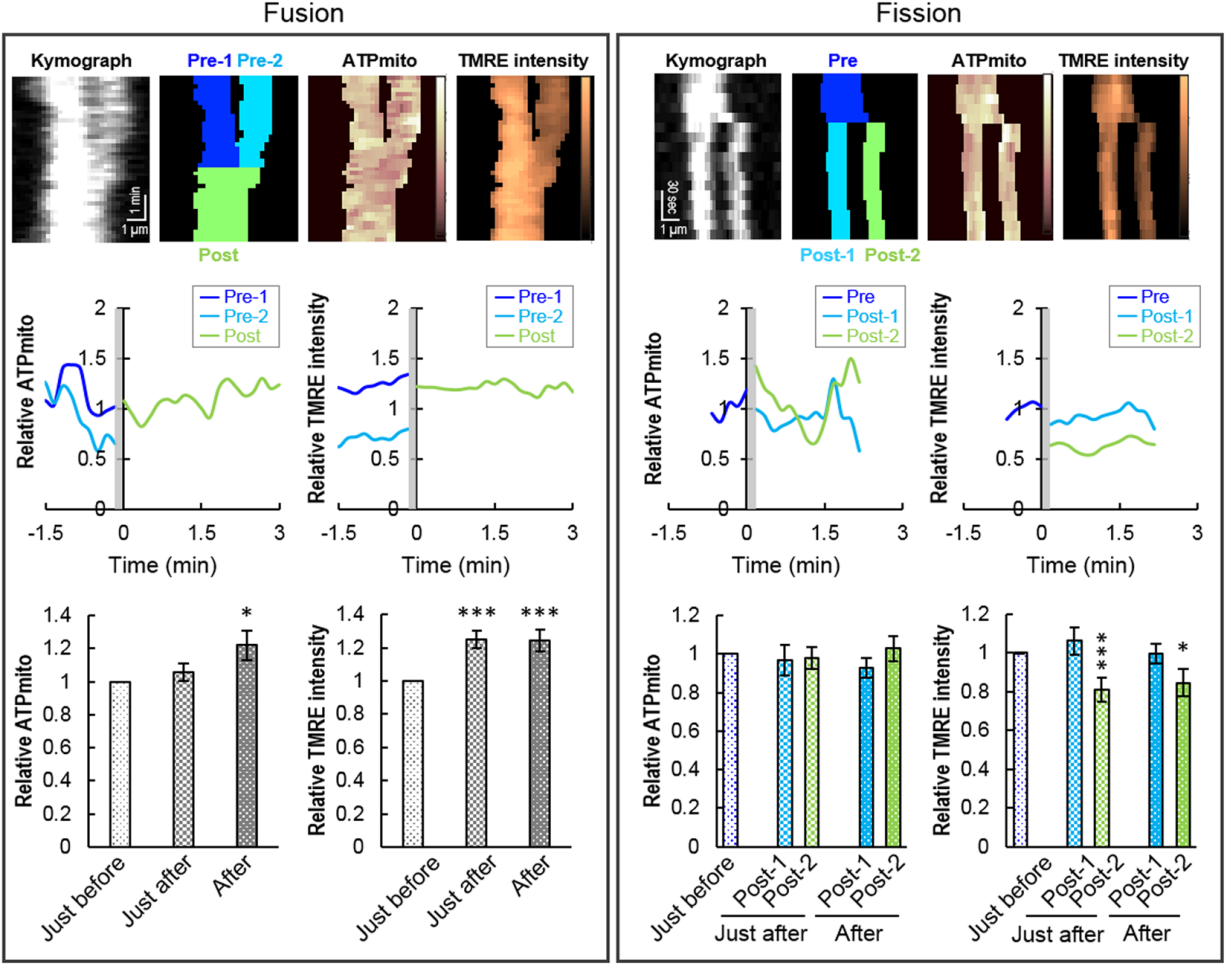
Change in mitochondrial ATP and inner-membrane potential during fission or fusion events. Typical kymograph showing the change in mitochondrial ATP and TMRE intensity during a mitochondrial (upper, left) fusion or (upper, right) fission event. Each colour table is linear and covers the full range of the data. Typical mitochondrial ATP levels or TMRE intensity behaviour (middle, left and right) visualised in the upper panels is demonstrated. Comparison of change in mitochondrial ATP levels and TMRE intensity just before (−1 to 0 min) and just after (0 to 1 min) or after (1 to 2 min) 20 fission and 20 fusion events (lower, left and right). Error bars represent SEM.

In addition to transported mitochondria, some mitochondria underwent fusion or fission events during observations. Using kymograph, we explored ATP_mito_ and IMP_mito_ behaviour during fusion or fission events. During fusion events, IMP_mito_ more polarized in a post-fusion mitochondrion compared to the average of the two pre-fusion mitochondria (Fig. 2, left). Likewise, ATP_mito_ in a post-fusion mitochondrion were higher than the average of the two pre-fusion mitochondria (Fig. 2, left). During fission events, there was a difference in IMP_mito_ between the two post-fission mitochondria. Then, we compared the changes in IMP_mito_ and ATP_mito_ in both of the two post-fission mitochondria, respectively: one with a relatively highly polarized and the other with a relatively less polarized IMP_mito_ (named Post-1 and Post-2 in Fig. 2, right, respectively). During fission events, the IMP_mito_ of Post-2 was less polarized than that of the pre-fission mitochondrion as previously reported^33^, while that of Post-1 was the same as that of a pre-fission mitochondrion (Fig. 2, right); however, no ATP_mito_ changes were observed. This result also indicated that ATP_mito_ and IMP_mito_ are not always behave similarly.

### IMP_mito_ and ATP_mito_ in distribution

**Figure 3:**
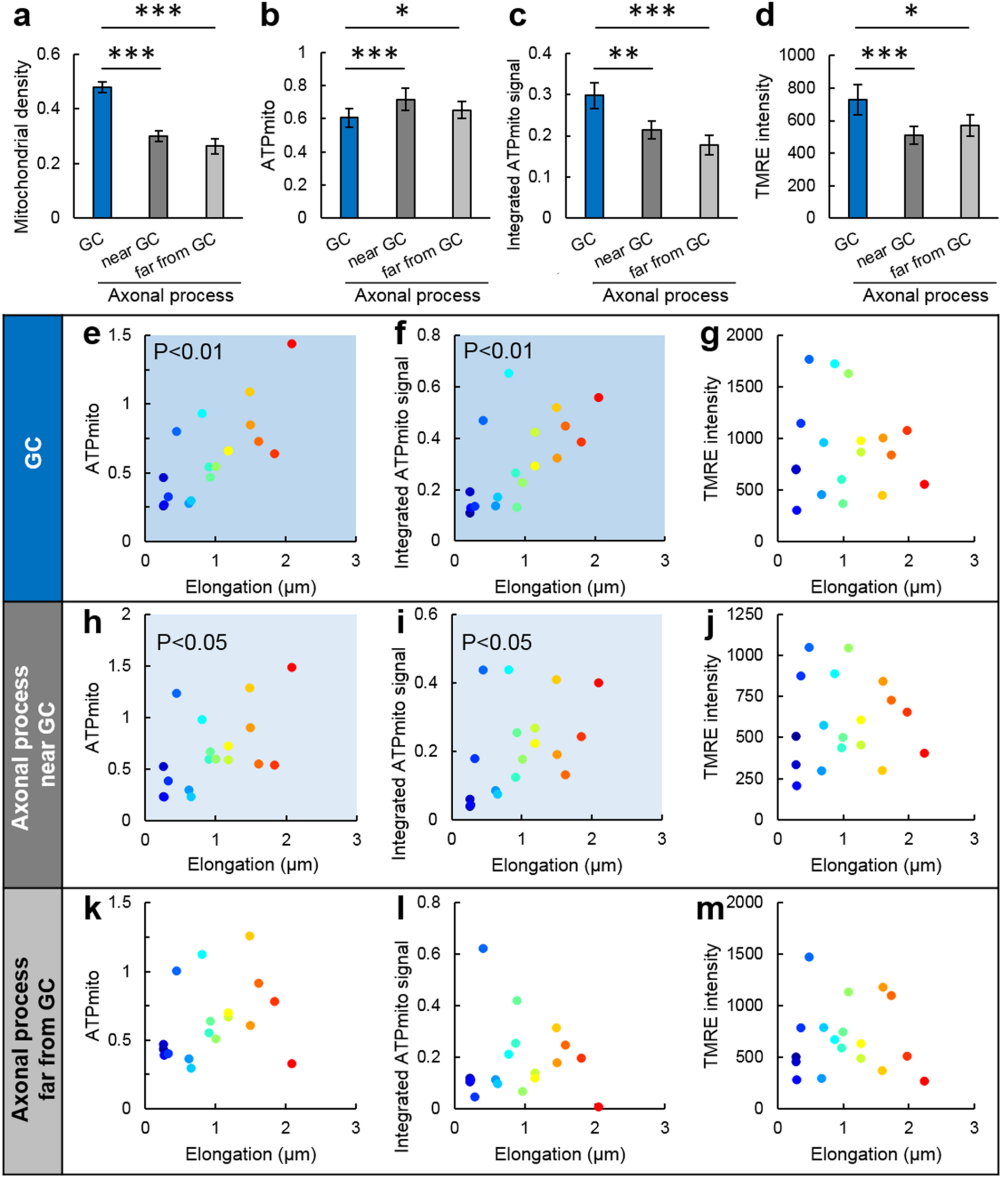
Mitochondria in GCs and axonal processes show different correlations between axonal elongation and mitochondrial ATP or inner-membrane potential. Average (**a**) density, (**b**) mitochondrial ATP levels, (**c**) integrated mitochondrial ATP signals (density × mitochondrial ATP levels), and (**d**) TMRE intensity of growth cones (GC; blue), axonal processes near GCs (0–20 µm from GC; grey) and axonal processes relatively far from GCs (20–40 µm from GC; light grey). Correlations between the distance of axonal elongation with (**e**,**h**,**k**) mitochondrial ATP, (**f**,**i**,**l**) integrated mitochondrial ATP signal or (**g**,**j**,**m**) TMRE intensity at (**e**–**g**) GCs, (**h**–**j**) axonal processes near GCs and (**k**–**m**) axonal processes relatively far from GCs. Data were taken from (**a**–**d**) 29 neurons or (**e**–**m**) a subset of 18 neurons grown from within those 29 neurons. In (**e**–**m**), each color represents each neuron. Error bars represent SEM.

We next assessed mitochondrial density using still images (Supplementary Fig. S4). Mitochondrial density was defined as: (sum of area dominated by all mitochondria)/(area size of GC or axonal process). We confirmed that mitochondrial density was higher in the GCs compared to the axonal process (Fig. 3a). Although average ATP_mito_ were slightly lower in the GCs compared to the axonal process, integrated ATP_mito_ signals (calculated by multiplying average ATP_mito_ and mitochondrial density) were high in the GCs due to the high mitochondrial density (Fig. 3b,c). Average IMP_mito_ relatively hyperpolarized in the GCs than in the axonal process (Fig. 3d).

### ATP_mito_ in GCs and axonal elongation have a positive correlation

Because GC is a structure related to axonal elongation, we quantified the distance of axonal elongation, and examined the role of mitochondrial dynamics in elongation. Among neurons grown during 10 min observation, the distance of axonal elongation and ATP_mito_ or integrated ATP_mito_ signals in the GCs showed a positive correlation (Fig. 3e,f). This correlation was stronger in GCs than in axonal processes (Fig. 3h,i,k,l). Furthermore, no correlation was found between elongation and IMP_mito_ for both GCs and axonal processes (Fig. 3g,j,m). These results suggest that ATP_mito_ in GCs, but not IMP_mito_ in GCs, contribute to axonal elongation.

### ATP_mito_ in GCs are involved in axonal elongation and GC morphological change

To verify the importance of ATP_mito_ in GCs for elongation, we further examined the effect of artificially disrupting IMP_mito_ or ATP_mito_ on axonal elongation using a mitochondrial uncoupler, carbonyl cyanide-p-trifluoromethoxyphenylhydrazone (FCCP), or the ATP synthase inhibitor, Oligomycin A, respectively.

Both FCCP and Oligomycin A induced apparent drawing back of neurons compared to the control condition (i.e., neurons without drug treatment; Supplementary Fig. S5a–c). However, the detail of change in IMP_mito_ and ATP_mito_ differed between FCCP and Oligomycin A, as well as between GCs and axonal processes. In GCs treated with FCCP, IMP_mito_ depolarized and ATP_mito_ decreased, and the declines correlated with axonal drawing backs (Supplementary Fig. S5d, upper). On the other hand, in axonal processes treated with FCCP, although IMP_mito_ depolarized, ATP_mito_ did not significantly change (Supplementary Fig. S5d, lower). As a result, no correlation was observed in axonal processes between the decrease in ATP_mito_ and axonal drawing back. Although drawing back and depolarization of IMP_mito_ showed a correlation in axonal processes, the peak was lower than that in GCs. After treatment with Oligomycin A, ATP_mito_ decreased both in GCs and axonal processes; however, IMP_mito_ levels did not change for either GCs or axonal processes (Supplementary Fig. S5e). Therefore, no correlation was found between IMP_mito_ and drawing back while there was a correlation between the decrease in ATP_mito_ and drawing back. Again, the correlation was more remarkable in GCs.

Axons drew back even when IMP_mito_ did not show depolarization (Supplementary Fig. S5c–e). Additionally, axonal drawing back correlated more with the decrease in ATP_mito_ than in IMP_mito_, and this correlation was higher in GCs than in the axonal processes. These results again indicated that ATP_mito_, especially in GCs, are crucial for axonal elongation.

In addition to axonal process drawing back, GC collapse was observed. To examine this change quantitatively, we conducted additional analyses of the changes in GC morphology, with a focus on the effect of Oligomycin A because it induced more drastic morphological changes than FCCP. The indices that we quantified were (i) area, (ii) newly appeared area, (iii) newly disappeared area, (iv) the sum of the newly appeared area and the newly disappeared area, (v) edge length and (vi) the ratio of edge length to area of both the peripheral- (P-) and the central- (C-) domains of the GC (Supplementary Fig. S6). The (ii) newly appeared area, (iii) newly disappeared area and (iv) their sum in the P-domain correlated positively with axonal drawing back after treatment with Oligomycin A. This means that these indices decreased along with drawing back of the axonal process. The (i) area of the C-domain and the (v) edge length of both the P- and C-domains exhibited peaks before (negative time lag values) and after (positive time lag values) drawing back. This means that axonal drawing back occurred after the C-domain area decreased (the positive peak at negative time lag), and that the C-domain area relatively increased after drawing back (the negative peak at positive time lag). Peaks in the (v) edge length and (vi) ratio of edge length to area of the P-domain shows that the P-domain attains a less protrusive morphology before axonal drawing back. The (v) edge length of the C-domain increases just after drawing back, which can be attributed to the crumpling of the C-domain when normal intact morphology collapses.

Moreover, correlation analysis between these indices and ATP_mito_ revealed that the (ii) newly appeared area, (iii) the newly disappeared area and (iv) their sum in the P-domain correlated positively with ATP_mito_ in the GC (Supplementary Fig. S7). Importantly, none of these indices correlated in the C-domain or the axonal process. These results indicate that although ATP_mito_ decrease and axonal drawing back were positively correlated (Supplementary Fig. S5e), analysis focused on GC morphology revealed that ATP downregulation manifested as less dynamic morphological changes in the P-domain of the GC. Because morphological changes in the P-domain relies mainly on actin dynamics, these results suggest the importance of ATP in actin turnover in GCs.

### Actin turnover is one processes related to neuronal ATP dynamics

To clarify the relevance of actin dynamics, we explored the changes in the GC morphology and cytosolic/mitochondrial ATP levels in response to treatment with Latrunculin A, an inhibitor of actin turnover (Supplementary Fig. S8). Latrunculin A treatment led to a decrease in the number of newly appeared area in the P-domain, but not the C-domain (Supplementary Fig. S8a). Latrunculin A treatment caused no change in ATP_mito_ (Supplementary Fig. S8d); however, cytosolic ATP levels increased both in the P- and C-domains (Supplementary Fig. S8b). Thus, this increase in cytosolic ATP is not because of enhancement of mitochondrial ATP, but may be because of suppression of actin turn over caused by Latrunculin A. Importantly, the increase in cytosolic ATP and the decrease in P-domain area were positively correlated (Supplementary Fig. S8c). The above indicates that actin turnover is a potential mechanism producing the correlation between ATP and neuronal dynamics.

### Correlation between ATP_mito_ and IMP_mito_

**Figure 4:**
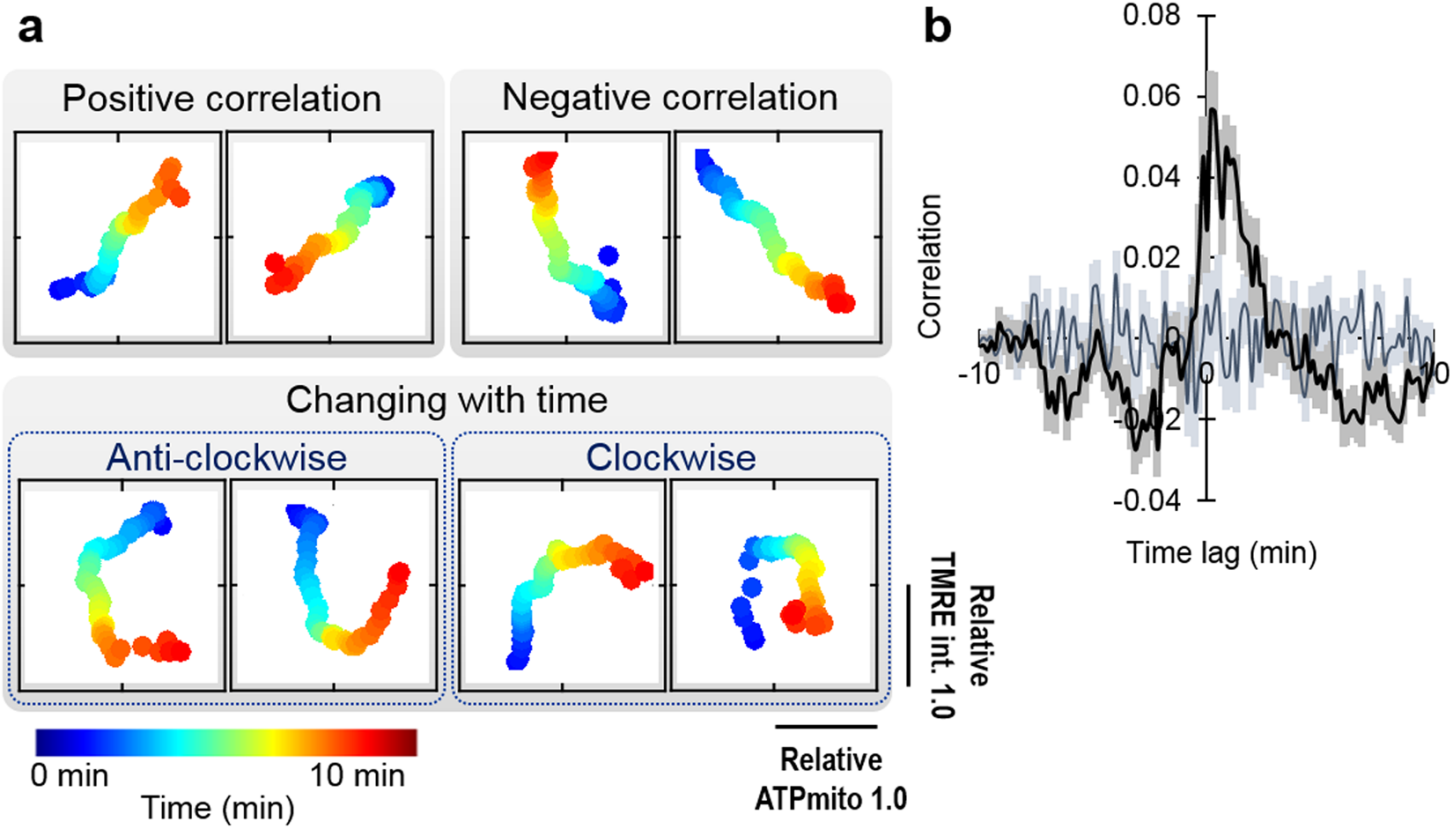
Mitochondrial ATP and inner-membrane potential do not always correlate. (**a**) Representive examples of transitions in TMRE intensity (vertical axis) and mitochondrial ATP levels (horizontal axis) in each mitochondrion. Time is represented by a pseudo-colour scale, which is linear and fully covers the data range. (**b**) Cross-correlation function between TMRE intensity and mitochondrial ATP levels. Average (black line) and SEM (grey bars) are indicated for 371 mitochondria from 50 axonal processes. The dark blue line and light blue soft-shaded bars represent the average and SEM of correlations calculated from randomly shuffled datasets, respectively.

Although ATP_mito_ and IMP_mito_ showed similar trends in some cases, different behaviour was found during transport, fission/fusion and axonal elongation. To investigate the relation of them in more detail, we visualised IMP_mito_ and ATP_mito_ over time (Fig. 4a). Each track was classified as (i) a positive correlation, (ii) a negative correlation, (iii) changing with time (drawing circle either clockwise or anti-clock wise), (iv) no correlation, or (v) other. Tracks classified as (iii) were further divided into eight parts by every 90 degree for both clockwise and anti-clockwise rotation directions. Here, clockwise rotation represents ATP_mito_ following IMP_mito_ and *vice versa* (Supplementary Fig. S9). Concurrently, cross-correlation functions between IMP_mito_ and ATP_mito_ were calculated. Conceptual wave-forms of the cross-correlation function are illustrated for (i), (ii), (iv), (v) and for the eight parts of (iii) in Supplementary Fig. S9. These results show that IMP_mito_ and ATP_mito_ are not necessarily correlated, although we cannot exclude the possibility that this is because of differences in the ability of the two probes to detect changes in each signal.

Considering the frequency of each type of events, it is understandable that an average of cross-correlation functions between ATP_mito_ and IMP_mito_ of all mitochondria showed a delayed positive peak (ATP_mito_ changed following IMP_mito_ change; Fig. 4b). The observed peak was statistically significant as randomly-shuffled data did not show a peak (Fig. 4b).

## Discussion

We conducted simultaneous imaging and spatiotemporal analyses of ATP_mito_ and IMP_mito_ during mitochondrial transport, fission/fusion and axonal elongation. We demonstrated that ATP_mito_ and IMP_mito_ are not always accurately correlated, and for axonal elongation, ATP_mito_ is a more dominant factor than IMP_mito_.

The role of mitochondria in axonal elongation has been reported previously; mitochondrial transport responds to axonal outgrowth or growth factors^34,35^, and the number and positioning of mitochondria is related to neurite elongation^8–10,13^. These reports suggest that mitochondrial localisation and ATP generation are crucial for axonal elongation. Although some of these previous studies measured cytosolic ATP levels directly or indirectly, no report has explored native ATP_mito_ under physiological conditions. In this study, we successfully demonstrated a correlation between ATP_mito_ within GCs with axonal elongation under physiological conditions.

Our results are also consistent with recent research demonstrating the importance of cytosolic ATP in the cellular motility of non-neuronal cells^28,36^. These studies explored cytosolic ATP, not ATP_mito_, possibly because these cell types have greater dependency on glycolysis compared to others^37,38^. Nevertheless, this study is one of the few that measured mitochondrial/cytosolic ATP itself to demonstrate their relevance in cellular morphological changes.

We have found that ATP_mito_ and IMP_mito_ are not necessarily correlated under physiological conditions. Some background phenomena may have contributed to this discrepancy. Firstly, differences in the ability of two probes to detect changes in each signal could obscure any potential correlation. Secondly, there is a possibility that the ATP_mito_ value reflects an accumulative IMP_mito_ value up to that time, instead of IMP_mito_ at the time, because IMP_mito_ at any given time is simply the difference in voltage in the mitochondrial inner membrane. On the other hand, ATP_mito_ has been considered to represent the net ATP pool in mitochondria at a given time. ATP_mito_ and summation of IMP_mito_ also positively correlated, and the correlation peak was highest when the IMP_mito_ summation period was approximately 2 min (Supplementary Fig. S10a). However, the 2 min period was an average, and the duration at which the strongest correlation was observed differed depending on each individual mitochondrion (Supplementary Fig. S10b). This may be because ATP_mito_ is a result of not only production, but also consumption and flux from the mitochondrial matrix. IMP_mito_ is generally considered an index linked to ATP_mito_ production, and the ATP_mito_ measured in this present study is a result of production and consumption/efflux. Thus, a decrease in the ATP_mito_ signal can be the result of either a reduction of ATP_mito_ production or an increase in ATP_mito_ consumption/efflux. This could also contribute to discrepancies in the correlation between the ATP_mito_ and IMP_mito_ signals.

Moreover, activities of electron transport chain (ETC) and ATP synthase activity are known to be flexible. Post-translational acetylation is one of the most representative modulations of mitochondrial activity. NDUFA9, an ETC complex I component, is known to be acetylated, which decreases ATP levels^39^. ATP synthase is also known to be acetylated^40^; however, this is only one facet of ATP synthase regulation, and ATP synthase activity is also under control of ADP inhibition^41^ or ATP synthase inhibitor (IF_1_)^42–44^.

In addition to direct effects on ETC or ATP synthase, there are other effectors that mediate IMP_mito_ and ATP_mito_ levels. The most well-known phenomenon involves proton leak *via* uncoupling protein (UCP)^45^, which diminishes the correlation between IMP_mito_ and ATP_mito_ by decreasing the number of protons that pass through ATP synthase.

Besides UCP-mediated uncoupling, mitochondrial permeability transition (MPT)^46^ is another major phenomenon caused by mitochondrial permeability transition pores (PTP)^46^. A PTP is a structure consisting of adenosine nucleotide translocase (ANT; translocator of ATP/ADP in mitochondrial inner membrane), voltage-dependent anion channel (VDAC; also called porin; a pore located at the mitochondrial outer membrane) and cyclophilin D^47^. The opening of PTP increases the permeability of mitochondrial membranes to molecules including proton, ATP and ADP^48^. Appropriate ATP/ADP translocation is essential for ATP_mito_ production in matrix, and MPT leads to loss of IMP_mito_; thus, opening of PTP would disrupt the balance between ATP_mito_ and IMP_mito_ under physiological conditions. Furthermore, MPT is known to be induced without PTP; rapid depolarization depending on the net translocation of protons from matrix to the intermembrane space has been reported in isolated mitochondria^49^.

Previous biochemical research on mammalian oxygen consumption by mitochondria in the standard state suggested that approximately 20% was uncoupled by mitochondrial proton leak and 80% was coupled to ATP synthesis^50^. Recently, a positive feedback mechanism that alters the response of IMP_mito_ to glucose concentrations was reported^51^, and the working condition of the mitochondrial uncoupling protein also differed with regards to IMP_mito_^52^. Clockwise or anti-clockwise trajectories of ATP_mito_ and IMP_mito_ also suggest that the relation between them is not linear, but rather influence each other, and the extent of the influence changes with time and condition.

In summary, we conducted simultaneous imaging of several mitochondrial properties in neurons. Detailed spatiotemporal image processing directly showed that the affect of native IMP_mito_ and ATP_mito_ are mutual; therefore, the correlation between the two properties changes over time under physiological conditions.

## Materials and Methods

### Experimental design

The sample size was not determined prior to experimentations. Pixels with an outlier value were excluded when calculating the average during image processing.

The number and composition of experiments are noted in figure legends. All data were statically evaluated by comparing with control conditions or random shuffled-datasets. Alternatively, when there was no control group, datasets were compared by suitable statistic tests (Refer to “*Statistical analysis*”).

### Plasmid

The mitAT1.03 plasmid^25,29,30^ was kindly provided by Professor Imamura (Kyoto University, Kyoto, Japan).

### Cell culture

Dorsal root ganglion neurons dissected from Wister rat embryos at embryonic day 18 were dissociated with 1 mg/mL trypsin (Sigma-Aldrich) in phosphate-buffered saline. The dissociated cells were cultured in Neurobasal medium without phenol red (Life Technologies), supplemented with 2% B27 (Life Technologies) and 50 ng/mL nerve growth factor (NGF; Alomone Labs). Cells were transfected with mitAT1.03 by electroporation and seeded on 35-mm glass-based dishes (IWAKI) double-coated with 0.01 mg/mL poly-D-lysine (SIGMA) and 10 mg/mL laminin (Invitrogen), and cultured at 37 °C in 5% CO_2_. Neurons transfected with mitAT1.03, an ATP_mito_ indicator, were also stained with tetramethylrhodamine ethyl ester (TMRE; Invitrogen), an IMP_mito_ indicator, and observed by confocal microscope 6–24 h after dissociation, coinciding with a period of axonal elongation (Supplementary Fig. S1).

All animal procedures were approved by the Ethics Committee of Keio University (permit number, 09106-(1)). In addition, all experiments were performed in accordance with relevant guidelines and regulations.

### Measurement of ATP_mito_ using mitAT1.03

Neurons were transfected with mitAT1.03 by electroporation using Neon (Life Technologies) just before plating. mitAT1.03 is a fluorescence resonance energy transfer (FRET)-based indicator, and is composed of the epsilon subunit of the F_o_F_1_-ATP synthase sandwiched by the cyan- and yellow-fluorescent proteins^25,29,30^. Additionally, this indicator accompanies the duplex of the mitochondrial targeting signal of cytochrome c oxidase subunit VIII, which is a protein that is normally localised to the mitochondrial inner-membrane^25^. FRET refers to a phenomenon between donor and acceptor molecules in which the energy from the excited donor is transferred to the acceptor when the emission spectrum of a donor overlaps the absorption spectrum of an acceptor. This phenomenon occurs when the two molecules are in close proximity, although the extent of the spectral overlap and orientation between the donor and the acceptor also affect FRET efficiency. The epsilon subunit assumes its folded form upon ATP binding, and is relaxed in the absence of ATP. As a result, changes in ATP levels can be estimated from the changes in FRET signal intensity, which is derived from alternation of the relative distance and orientation between the two fluorophores.

### Measurement of IMP_mito_ using TMRE

Neurons were stained 6–24 h after transfection with 25 nM TMRE for 10 min at 37 °C and imaged. TMRE was not removed after the initial staining; therefore, there was additional TMRE dye at 5 nM concentration in the cytoplasm and medium during observation. TMRE is a cell-permeant and positively-charged dye, which accumulates in the relatively negatively-charged mitochondria. Because the extent of TMRE incorporation depends on the extent of mitochondrial polarization, mitochondrial polarization could be estimated from TMRE fluorescence; higher TMRE intensity indicates a greater extent of IMP_mito_ polarization (hyperpolarization).

### Fluorescence microscopy

All fluorescent imaging experiments were performed using a confocal laser-scanning microscope (FV1000 IX81; OLYMPUS) with a ×60 oil immersion objective lens (Supplementary Figs S11, S12).

mitAT1.03 and TMRE were excited by a diode laser (440 nm) and a helium-neon laser (559 nm), respectively, through a 20/80 beam splitter. Emitted fluorescence was separated by dichroic mirrors (510 nm and 560 nm), and signals from mseCFP, mVenus and TMRE were detected at 460–500 nm, 510–530 nm and 575–675 nm, respectively, using band pass filters. The widths of the 460–500 nm and 510–530 nm filters were manually adjusted using monochromators to ensure the lowest amount of background and fluorescence leakage from other channels or excitation lights. Width of 575–675 nm was a specified value of a barrier filter in our system.

Images were acquired at regions approximately 0–150 µm from the edge of axons, with a resolution of 0.132 μm/pixel every 10 sec for 10–15 min. During imaging, cells were maintained at 37 °C and in a 5% CO_2_ atmosphere using a stage chamber (TOKAI HIT).

### Image processing and analysis of mitochondrial behaviour

Acquired images were processed using our novel image processing software (Supplementary Figs S13, S14). It was written in MATLAB, and developed for this study referring to previous researches^27,28^.

Acquired fluorescent images were median filtered (3 × 3) and the background was subtracted. The background-subtracted images were again median filtered (3 × 3).

Next, to obtain signals from each fluorescent proteins or dye, linear-unmixing processing was conducted for each pixel (refer to Supplementary Fig. S13 and the following paragraph titled “*Unmixing processing*” for detail procedure).

After linear-unmixing, images were again median filtered (3 × 3). Then, merged images of mseCFP and mVenus from mitAT1.03 were aligned in order of time to obtain a kymograph. A kymograph based on TMRE fluorescence was also produced. Each mitochondrial track was manually identified while referring to the both kymographs. This double check enhanced the certainness of mitochondrial detection. Position, moving direction, displacement and velocities of each mitochondrion for each time point was calculated based on the tracks. When calculating mitochondrial displacement and velocity, axonal elongation was considered. The curve of the axonal process was also corrected for the estimation of displacement and velocity. The time course of each IMP_mito_ and ATP_mito_ was estimated from averaged TMRE intensities or from the pixel-by-pixel value of mVenus/mseCFP of mitAT1.03 within a mitochondrion, respectively.

### Unmixing processing

Cells labelled with cytosolic CFP, cytosolic Venus or TMRE were prepared and excited under experimental filter, laser and detection wavelength conditions (Supplementary Fig. S13). The acquired images were processed with background noise reduction and median filtration. Then, the cellular (for CFP- or Venus-expressing cells) or mitochondrial area (for TMRE-labelled cells) was automatically detected in each channel (Ch. 1, CFP-expressing cells; Ch. 2, Venus-expressing cells; Ch. 3, TMRE-stained cells). Relative leakage matrix was derived by quantifying the average fluorescence from the detected areas in each channel. Finally, intrinsic signals were obtained by multiplying the inverse matrix of the leakage matrix to the acquired signals pixel-by-pixel.

### Image processing and analysis of neuronal behaviour

Axonal elongation and drawing back after Oligomycin A or FCCP treatment were estimated by identifying neck position of the GC as a landmark in DIC (differential interference contrast) images.

The neck position is a boundary point between GC and the axonal process. Because the width of the axonal process is mostly constant, the point where the width increases compared to the adjacent point was determined as the neck position. The GC neck position was visually detected in every third image, and automatically complemented for the remaining images. Axonal elongation and drawing back distances were quantified from the displacement of two neck positions. The distance of axonal elongation is a displacement of the neck positions for 10 min observation. The distance of drawing back was defined as the distance over which the GC neck position moved back at 10 minutes after drug treatment.

The software used to analyse the DIC images was originally developed for this study; however, the main procedure that utilised the manually-detected neck position as a landmark of axonal elongation was based on a previously published protocol^27^.

### Distinguishing transport categories

The novel software used to generate kymographs was developed for the purposes of this study.

Mitochondria with an average velocity larger than |±0.5 × 10^−2^| µm/sec were classified as moving mitochondria. However, some mitochondria showed not only a move status but also a pause status during the observation; mitochondria displaced more than |±1| µm were also considered as moving mitochondria. Mitochondria other than moving mitochondria were classified as stationary mitochondria. Anterograde or retrograde transport was classified based on the direction of movement. Here, velocity of a mitochondrion was estimated as (displacement distance from first position to final position during an observation [µm])/(time of the observation [sec]), even mitochondria showed bidirectional movements. Therefore, the velocity of a mitochondrion that stopped during movement or moved bidirectionally was calculated to be slower. Other parameters such as ATP_mito_ or IMP_mito_ of a mitochondrion were calculated by averaging all time flames of which the mitochondrion was detected. For this reason, mitochondria moving across each other during the observation were excluded when deriving Figs 1, S2, S3 and S9.

### Analysis of fusion and fission events

Fusion or fission events were manually identified by referring to the corresponding kymographs and movies; kymographs provide information related to mitochondrial dynamics or the time course of ATP_mito_ or IMP_mito_, and movies enable us to differentiate between a fusion/fission event and the overlapping/separation of two mitochondrial moving across each other. Events found in the kymograph that could not be confirmed as fission/fusion using the corresponding movies were excluded from the analysis in Fig. 2. During fusion events, the average IMP_mito_ or ATP_mito_ levels of two pre-fusion mitochondria were compared with those of a post-fusion mitochondrion. During fission events, the IMP_mito_ or ATP_mito_ levels of two post-fission mitochondria were compared with those of a pre-fission mitochondrion.

### Visualising the correlation between ATP_mito_ levels and IMP_mito_

We applied moving averages (1.5 min before and after) to the relative ATP_mito_ and IMP_mito_, and demonstrated the time transition of the values using pseudo colour.

### Correlation analysis and random shuffling

The cross-correlation function was calculated using a MATLAB function after smoothing and normalising the original sequential data. As a control, random-shuffled datasets were generated from the original datasets, and the correlation between the two control datasets was calculated.

### Comparison of mitochondrial properties in the GC and axonal process

Mitochondrial density, IMP_mito_ and ATP_mito_ of Fig. 3 were derived from a single image that correspond to the first image of a time-lapse observation. Additionally, the morphology of the axonal process was detected using the first DIC image. Distances of axonal elongation were calculated by comparing the first and the last DIC image. Density was calculated as a ratio: (sum of area dominated by all mitochondria)/(area size of GC or axonal process). ATP_mito_ and IMP_mito_ were calculated from the average of all mitochondria included in the area of interest. The integrated ATP_mito_ signal was estimated as: (density) × (ATP_mito_). The widths of axonal process regions (20 µm) were decided so as to have the same area as that of GCs.

### Inhibitor experiments

FCCP (final concentration, 5 µM; SIGMA) or Oligomycin A (final concentration, 2 µM; SIGMA) were applied onto a glass-based dish at 5 min of the whole 15 minute observation. To evaluate changes in ATP_mito_ or IMP_mito_ after application of FCCP or Oligomycin A, the average values before and after application were compared. In Supplementary Fig. S5c, the drawing back distance of neck positions after drug treatment was compared with the displacement of the neck positions of control neurons for 10 min under physiological conditions.

### Quantification of morphological change in growth cone

In Supplementary Figs S6–S7, morphology of growth cone was quantified by semi-automatic analysis generated by our own for this study. Briefly, growth cone morphology of every single image was manually detected for all time flames. In addition, morphology of C-domain and GC neck position were defined by manually for all these images. P-domain was defined by subtracting C-domain area from GC area. From these morphologies, area and edge length were calculated for both P-domain and C-domain, respectively. Newly appeared area and newly disappeared area were quantified by comparing the two sequential images.

### Imaging of cytosolic ATP level

In Supplementary Fig. S8, neurons expressing cytosolic ATP sensor AT1.03^25^ were imaged. The AT1.03 plasmid was also provided by Professor Imamura (Kyoto University, Kyoto, Japan). Cytosolic ATP levels were estimated by calculating mVenus/mseCFP ratio. GC morphology was defined from fluorescence of mseCFP + mVenus automatically. Procedures of these image processing were based on previous study^27^. Latrunculin A (TOCRIS) was applied at the final concentration of 100 nM.

### Statistical analysis

Data were evaluated using the Mann-Whitney U-test or Wilcoxon signed-rank test. When more than three groups were compared, Bonferroni correction was performed. As for Fig. 3e–m, Spearman’s rank correlation coefficient was used. Asterisks (***, **, *) indicate a significance of <0.001, <0.01 and <0.05, respectively.

### Data Availability

The datasets generated and analysed during the current study are available from the corresponding author on reasonable request.

## Electronic supplementary material

Supplementary Information
