# Supplementary material for: Transitional correlation between inner-membrane potential and ATP levels of neuronal mitochondria
Source: Sci Rep. 2018 Feb 14;8:2993. doi: 10.1038/s41598-018-21109-2 (PMC5813116; doi:10.1038/s41598-018-21109-2)
Supplement: Supplementary file 1 — Supplementary Information [file 41598_2018_21109_MOESM1_ESM.pdf]

Transitional correlation between inner-membrane potential and ATP levels of neuronal mitochondria

R. Suzuki, K. Hotta, K. Oka

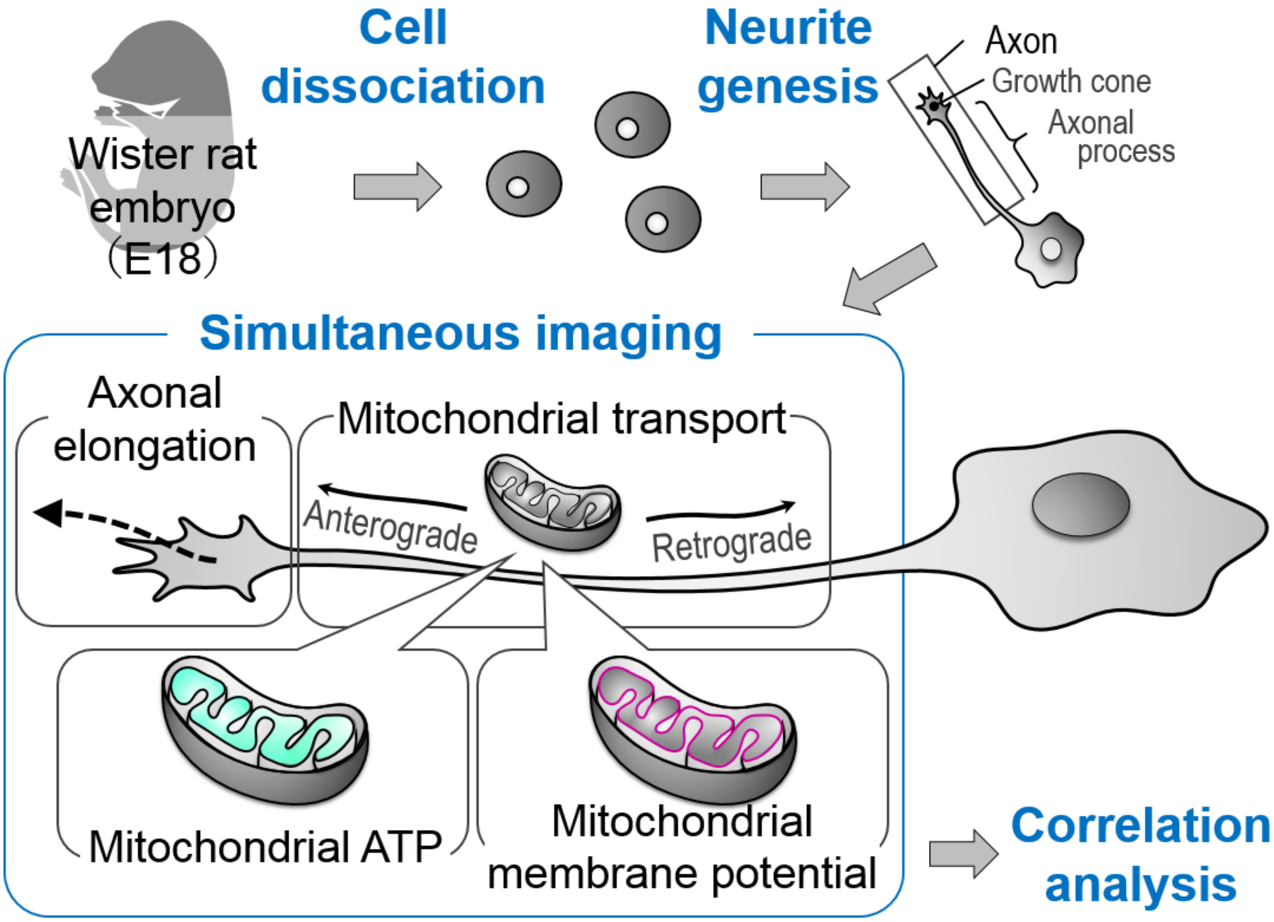

Supplementary Figure 1. Experimental procedure of the study.
We dissociated and cultured dorsal root ganglion neurons from day 18 rat embryos and observed their mitochondrial dynamics along with axonal elongation.

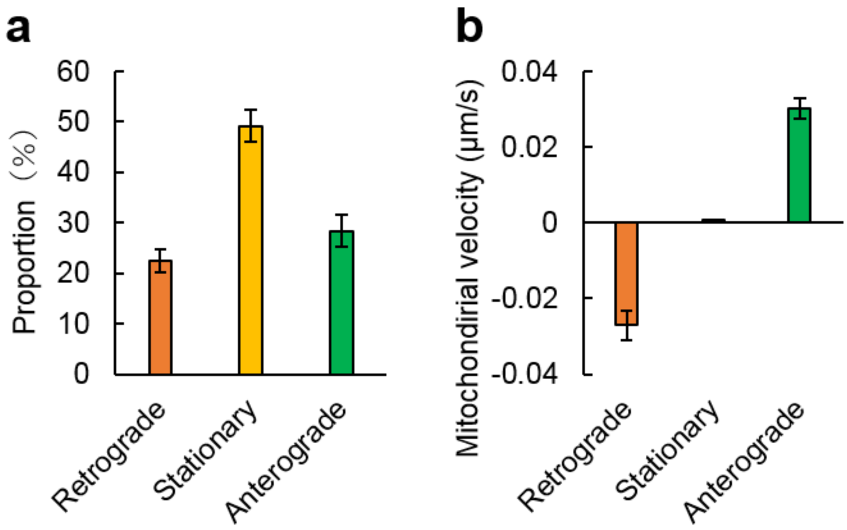

Supplementary Figure 2. Detail characterization of mitochondria. Average mitochondrial (**a**) proportions, and (**b**) velocity for 33 retrograde, 78 stationary, and 27 anterograde mitochondria from 50 neurons. Error bars represent SEM.

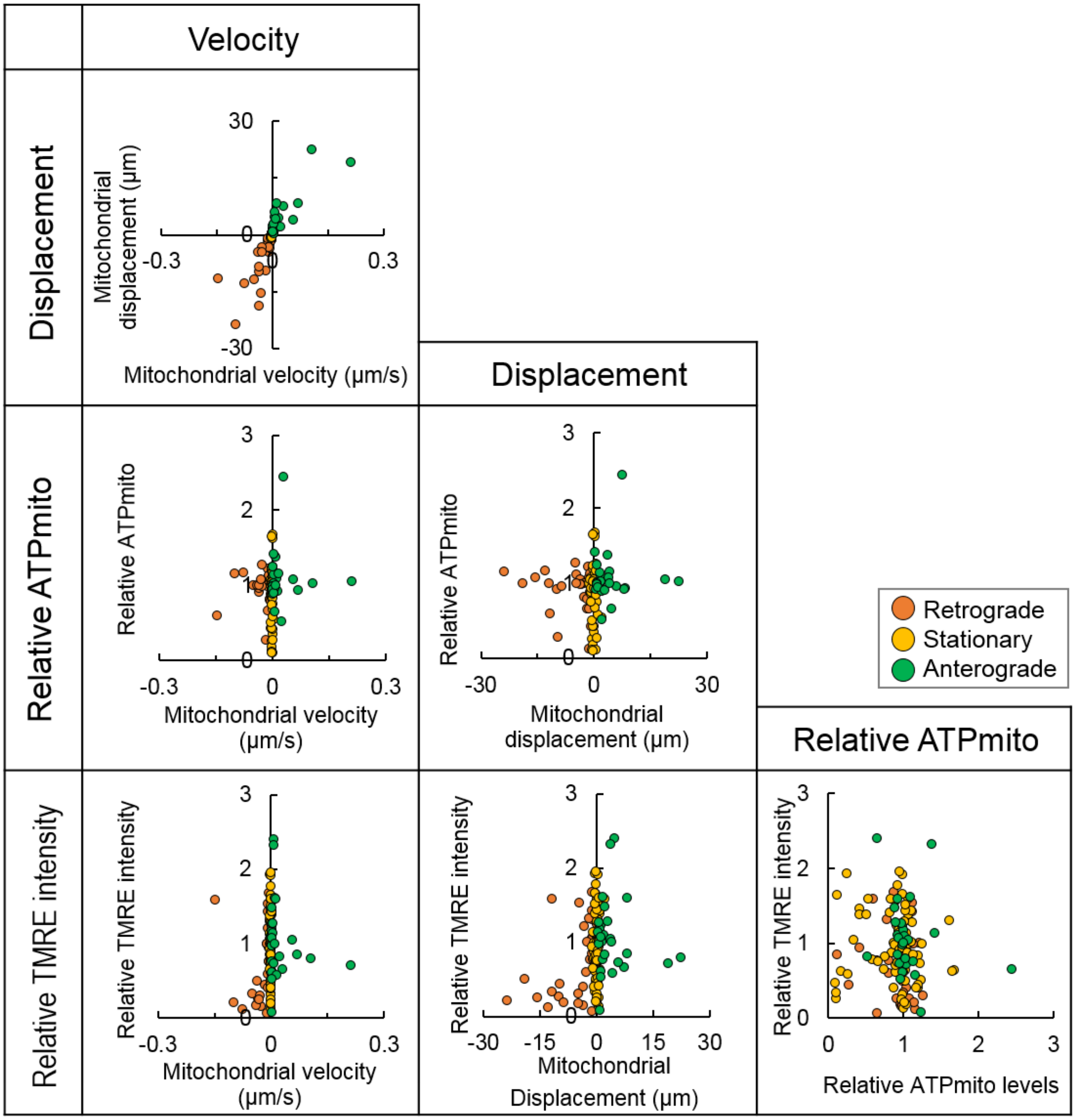

Supplementary Figure 3. Correlation between parameters characterizing mitochondria.

Correlations of mitochondrial velocity, displacement, relative mitochondrial ATP, and relative TMRE intensity were investigated. No obvious correlation was found except the natural correlation between velocity and displacement.

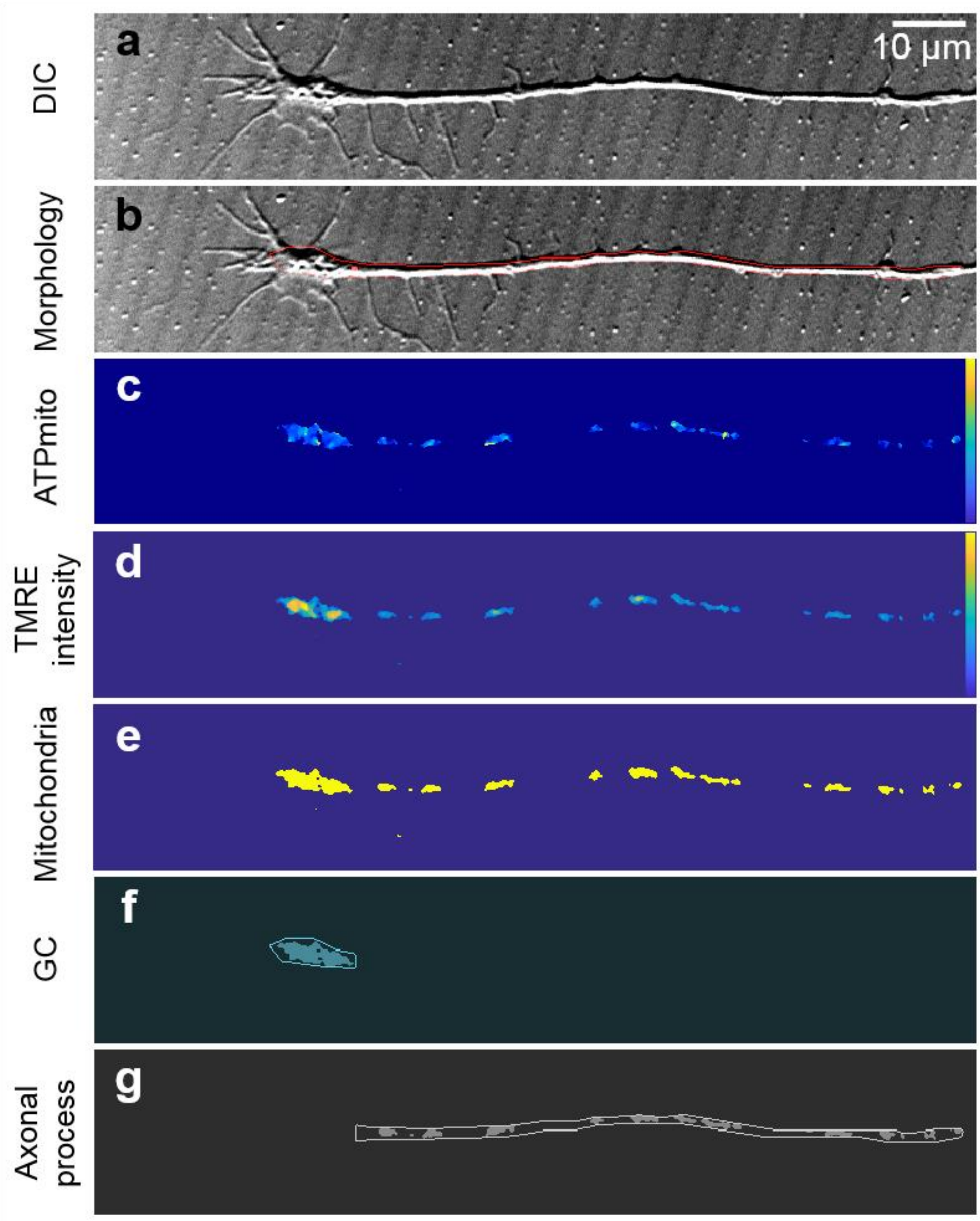

Supplementary Figure 4. Comparison of mitochondrial properties between GC and axonal process using still images.
(**a**) DIC image of a typical neuron. (**b**) Red line indicates detected morphology of growth conal central (C-) domain and axonal process. A neck of GC represented by a red point was manually defined. Pseudo color image of (**c**) mitochondrial ATP levels and (**d**) TMRE intensity. (**e**) Detected mitochondria. Morphology of the area and mitochondrial location within the are indicated for both (**f**) GC and (**g**) axonal process.

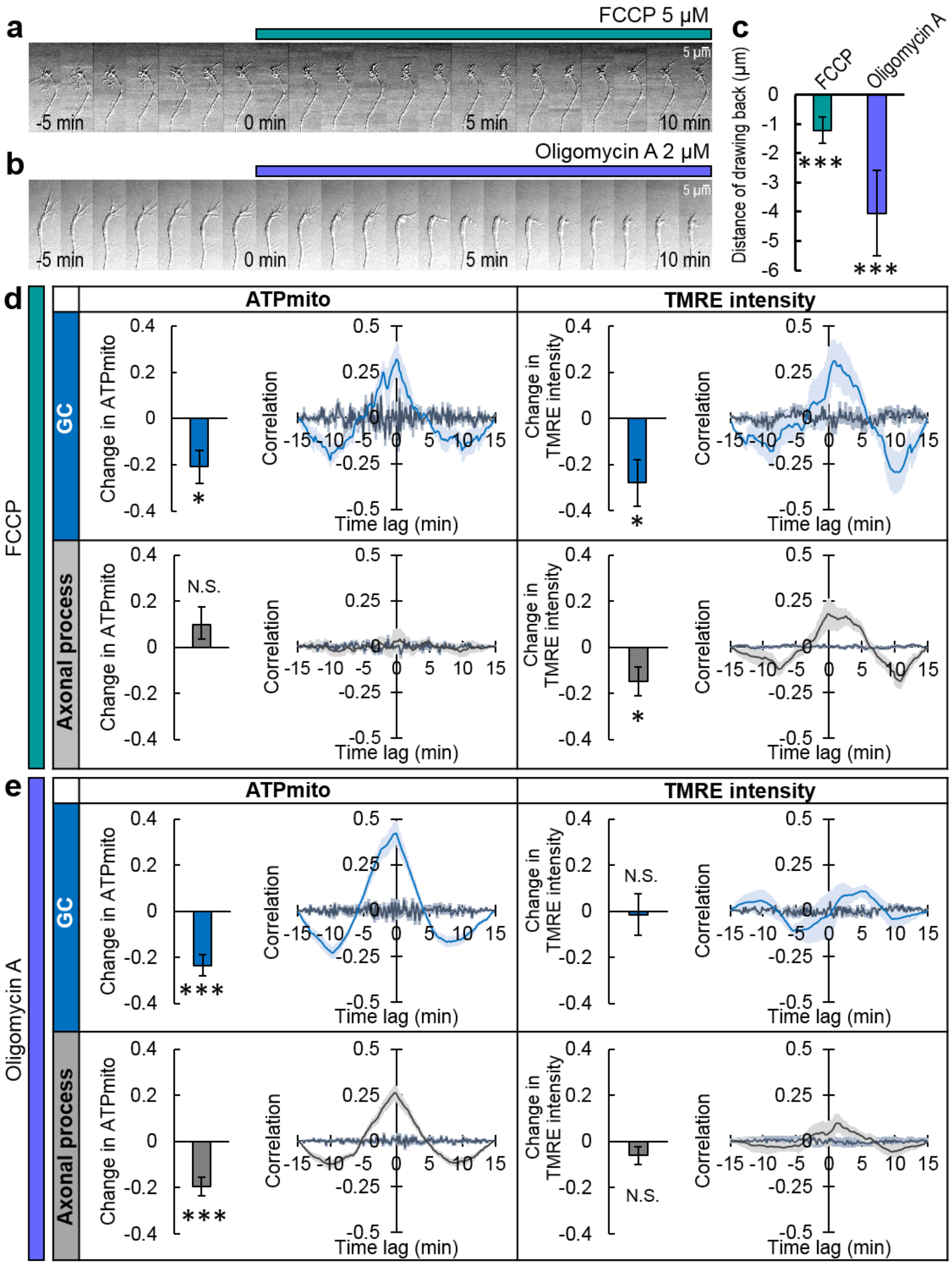

Supplementary Figure 5. Mitochondrial ATP in GCs had a correlation with axonal dynamics.

Typical images of axons treated with (**a**) 5 μM FCCP or (**b**) 2 μM Oligomycin A. (**c**) Average distance of axonal drawing back after treatment of FCCP or Oligomycin A. Average changes and cross-correlation functions with drawing back of (left) mitochondrial ATP levels or (right) TMRE intensities when axons were treated with (**d**) FCCP (7 axons) or (**e**) Oligomycin A (11 axons). In figures of average changes, error bars represent SEM. In figures showing cross-correlation functions, blue lines (in upper rows), grey lines (in lower rows), and dark-blue lines (in all figures) represent correlations calculated in GCs, in axonal processes, and from random-shuffled datasets, respectively. Light-shaded bars attached to the lines represent SEM.

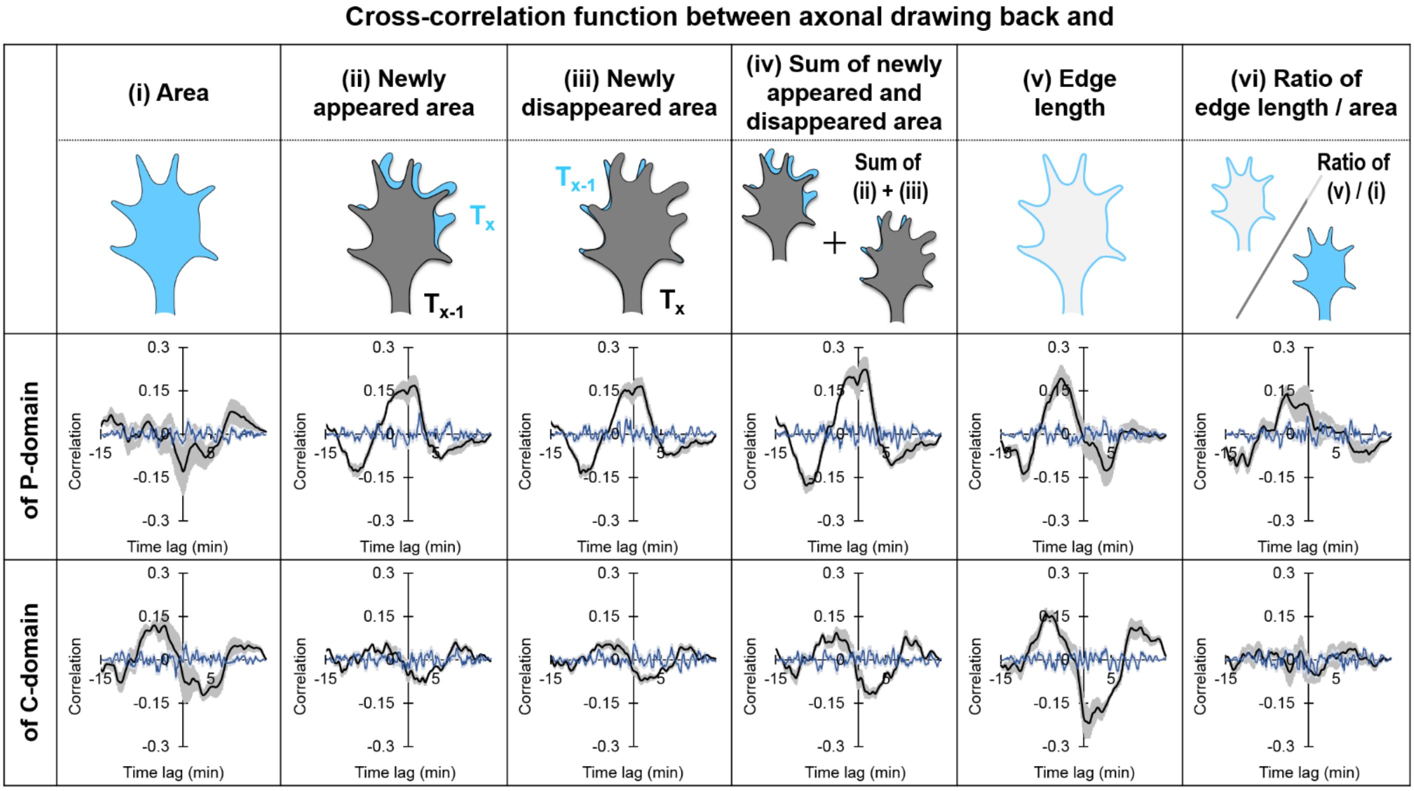

Supplementary Figure 6. Correlation between changes in GC morphology and axonal process dynamics.
Cross-correlation function between neck position drawing back and each index of GC morphology (black lines). Blue lines represent cross-correlation function derived from randomly shuffled datasets. Both the black and blue lines are the averaged results from 11 neurons. The horizontal axis represents time lag (min), and the vertical axis represents correlation. Light-shaded bars attached to the lines represent SEM.

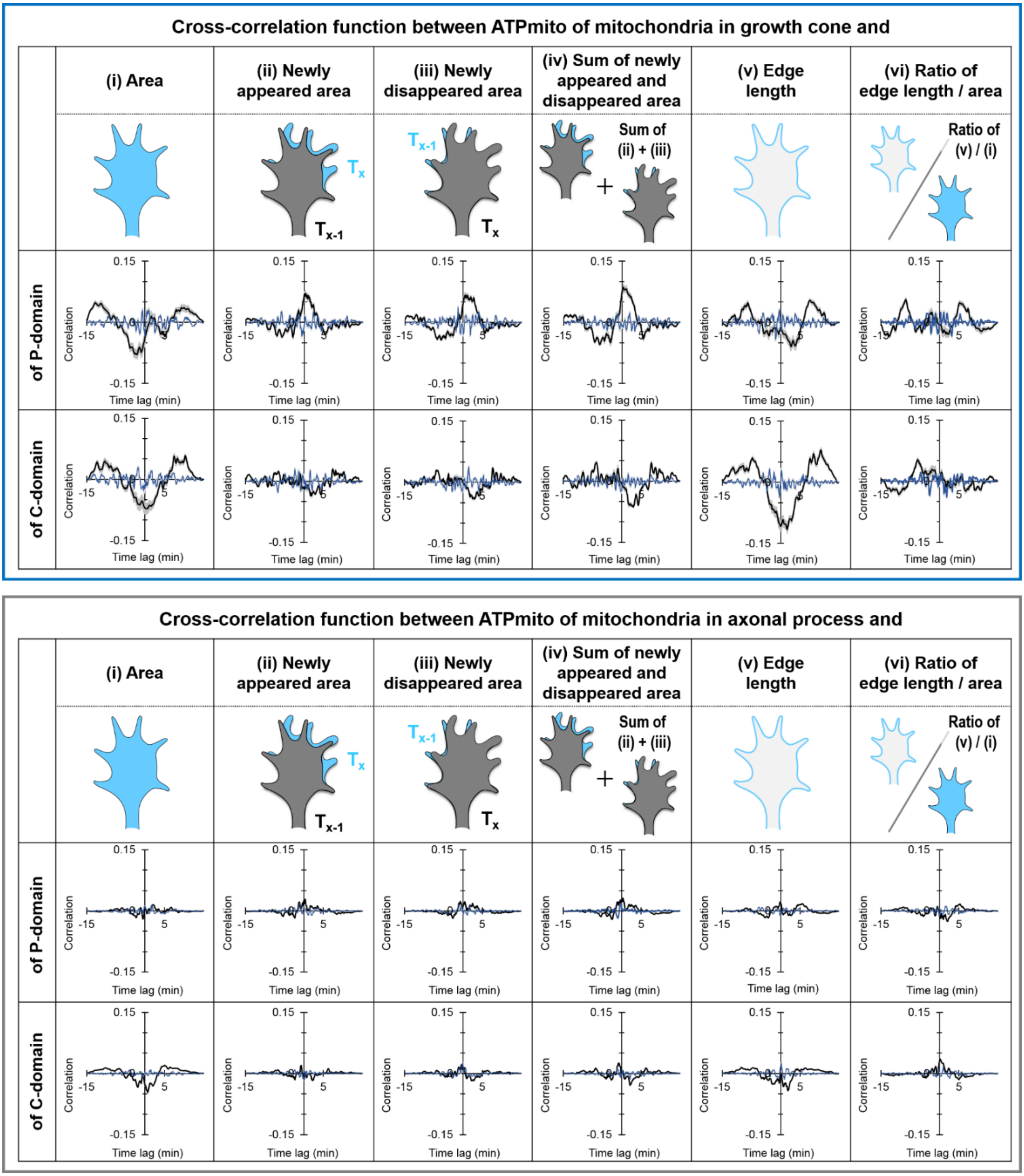

Supplementary Fig. 7. Correlation between changes in GC morphology and mitochondrial ATP.

Cross-correlation function between changes in mitochondrial ATP and each index of GC morphology (black lines) in either GC mitochondria (upper) or axonal process mitochondria (lower). Blue lines represent cross-correlation function derived from randomly shuffled datasets. Both the black and blue lines are averaged results from 11 neurons. The horizontal axis represents time lag (min), and the vertical axis represents correlation. Light-shaded bars attached to the lines represent SEM.

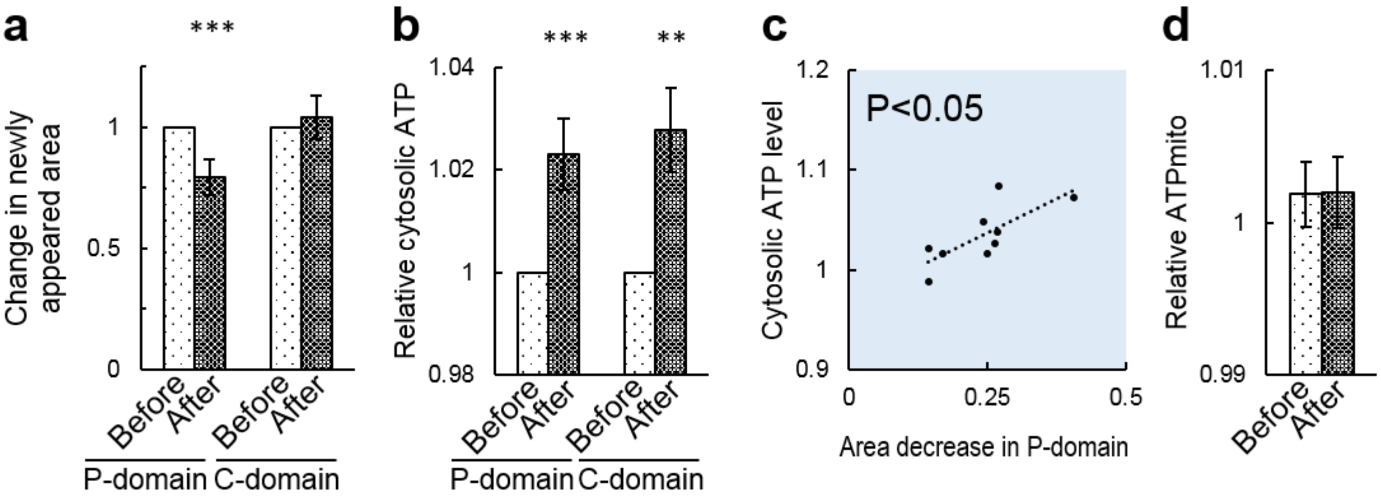

Supplementary Figure 8. Effects of inhibition of actin turnover on GC dynamics.
(**a**, **b**) Quantitative analyses of changes in (**a**) newly appeared area and (**b**) cytosolic ATP levels before and after treatment with Latrunculin A (n = 9). Latrunculin A decreased newly appeared areas in only the P-domain, whereas cytosolic ATP levels increased in both the P- and C-domains. (**c**) Correlation between the degree of decrease in the P-domain area and the increase in cytosolic ATP level in GCs. (**d**) Comparison of mitochondrial ATP before and after Latrunculin A treatment (n = 5). Mitochondrial ATP was not altered by Latrunculin A treatment. Error bars represent SEM.

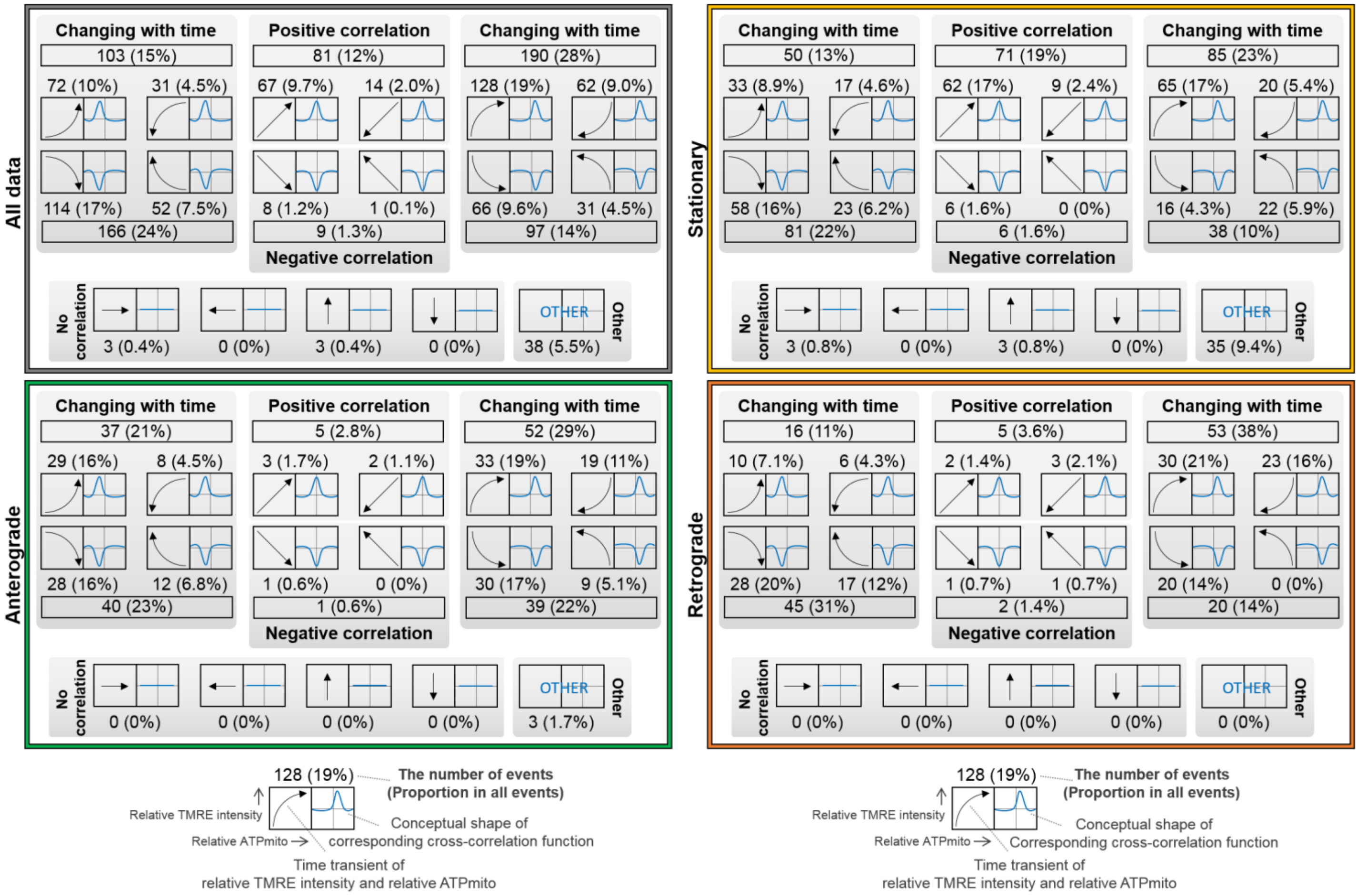

Supplementary Figure 9. Detailed trajectories of TMRE intensity and mitochondrial ATP levels.

Illustrated time transients, the conceptual shapes of corresponding cross-correlation functions, and the numbers and proportions of each type of mitochondrial track from 50 neurons.

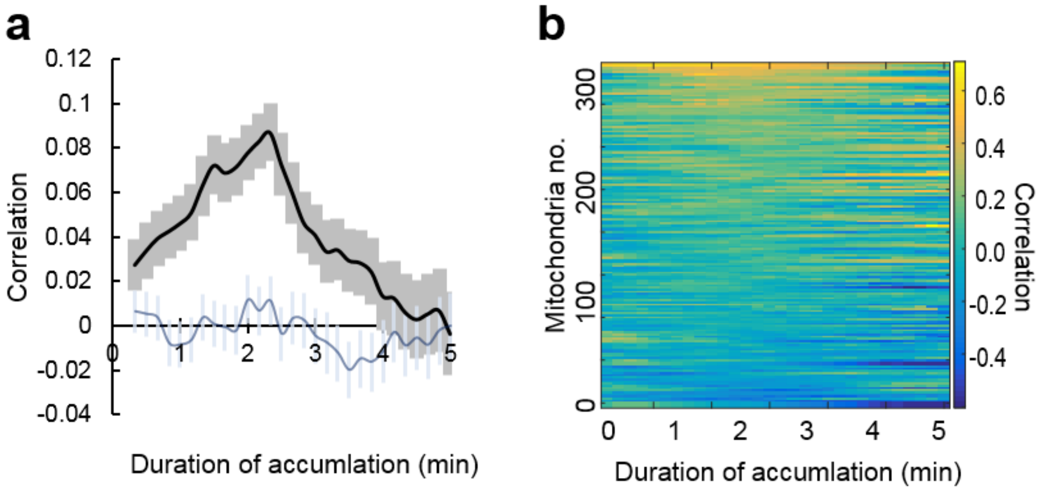

Supplementary Figure 10. Detailed analysis of mitochondrial ATP and TMRE intensity.

(**a**) Cross-correlation function between accumulative TMRE intensities and mitochondrial ATP. A blue line represents cross-correlation function derived from randomly shuffled datasets. Light-shaded bars attached to the lines represent SEM. (**b**) Details of the correlation analysis. The width of the time period with the strongest correlation between mitochondrial ATP and accumulative TMRE intensity differ in each individual mitochondrion.

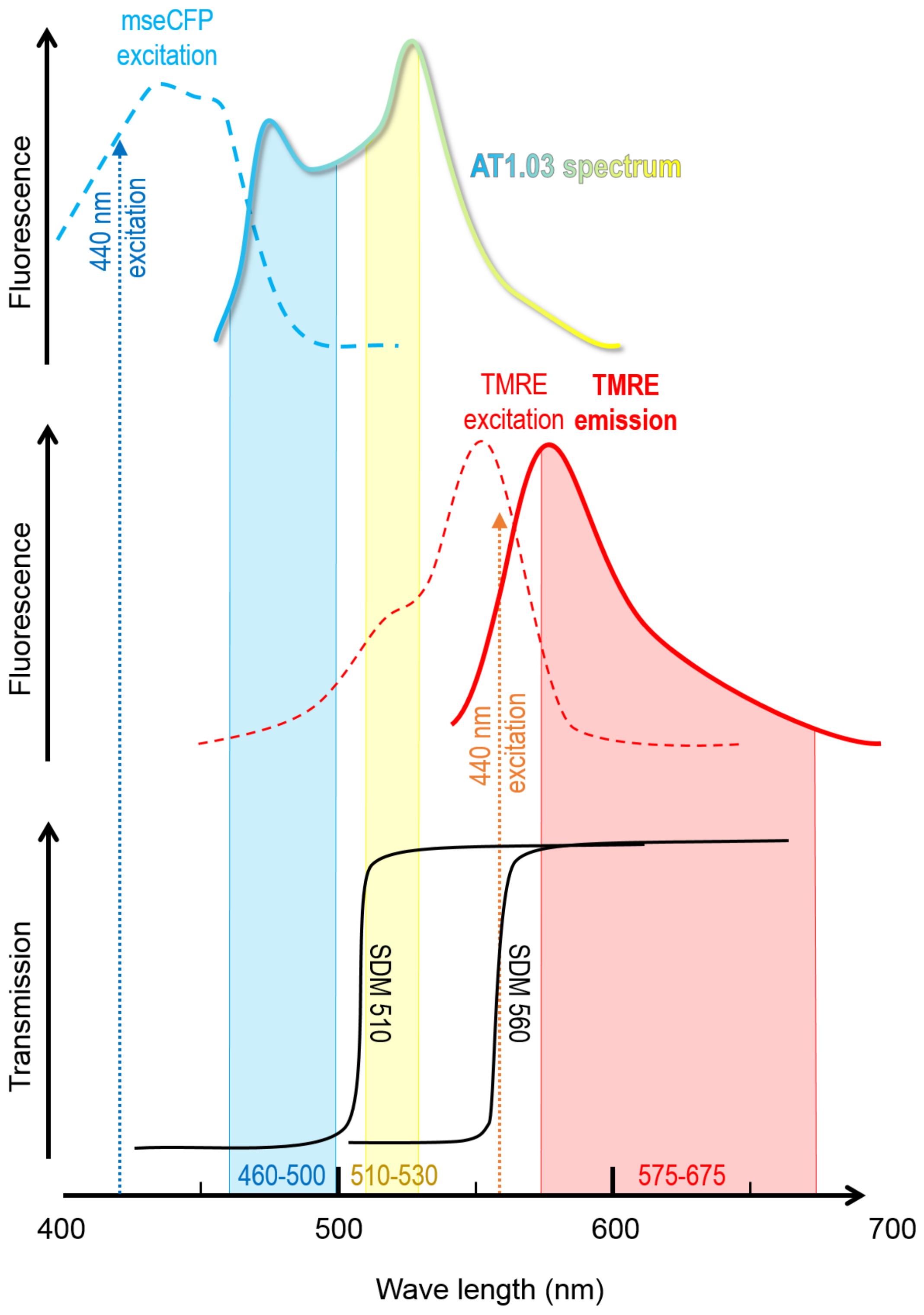

Supplementary Figure 11. Schematic representation of the fluorescence character of probes and appropriate optical setups.

SDM is an abbreviation for signal dichroic mirror (refer to Supplementary Fig. S12). Source: Imamura et al., 2009; Yamanaka et al., 2015; Chalmers and McCarron, 2008.

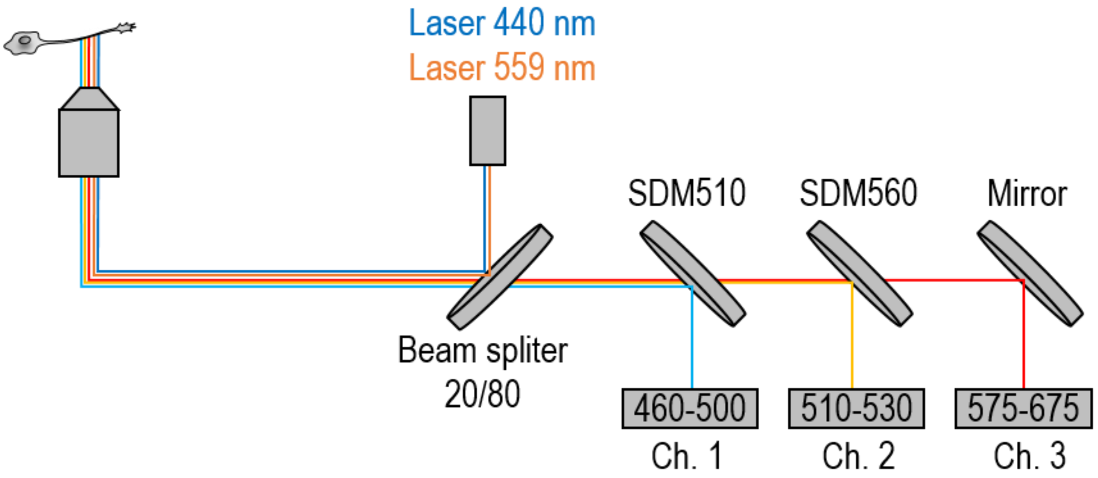

Supplementary Figure 12. Schematic representations of microscope setup.
SDM is an abbreviation for signal dichroic mirror. 460–500, 510–530 and 575–675 represent the wavelength (in nm) of band pass filters.

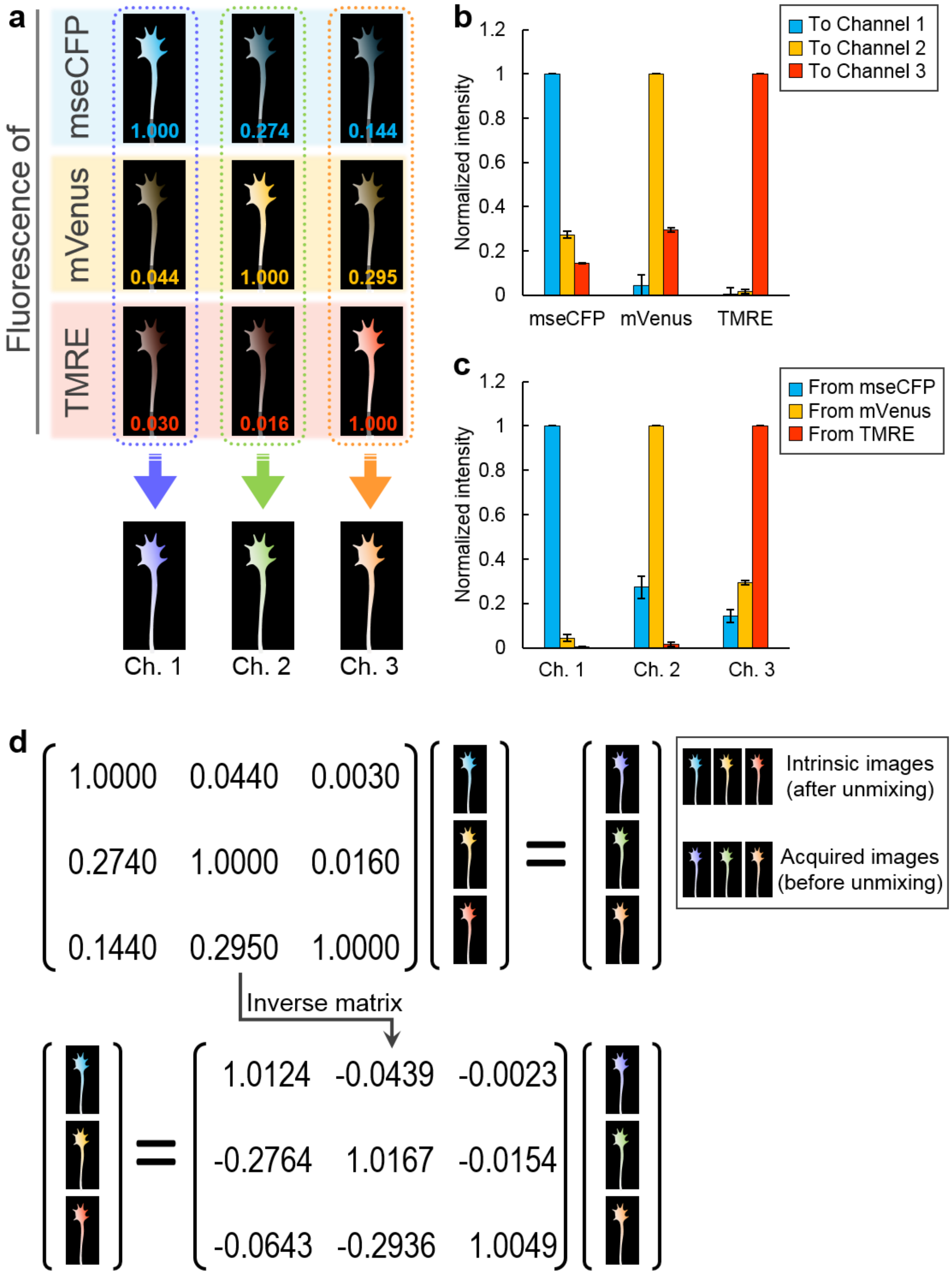

Supplementary Figure 13. Unmixing processing procedure.
(**a**) Schematic image of fluorescent leakage. Values attached to each image represent the relative intensity in each channel. (**b**) Fluorescent leakage into each channel. (**c**) Fluorescent components in each channel. (**d**) Schematic representation of the procedure for unmixing calculation. Error bars represent standard deviation.

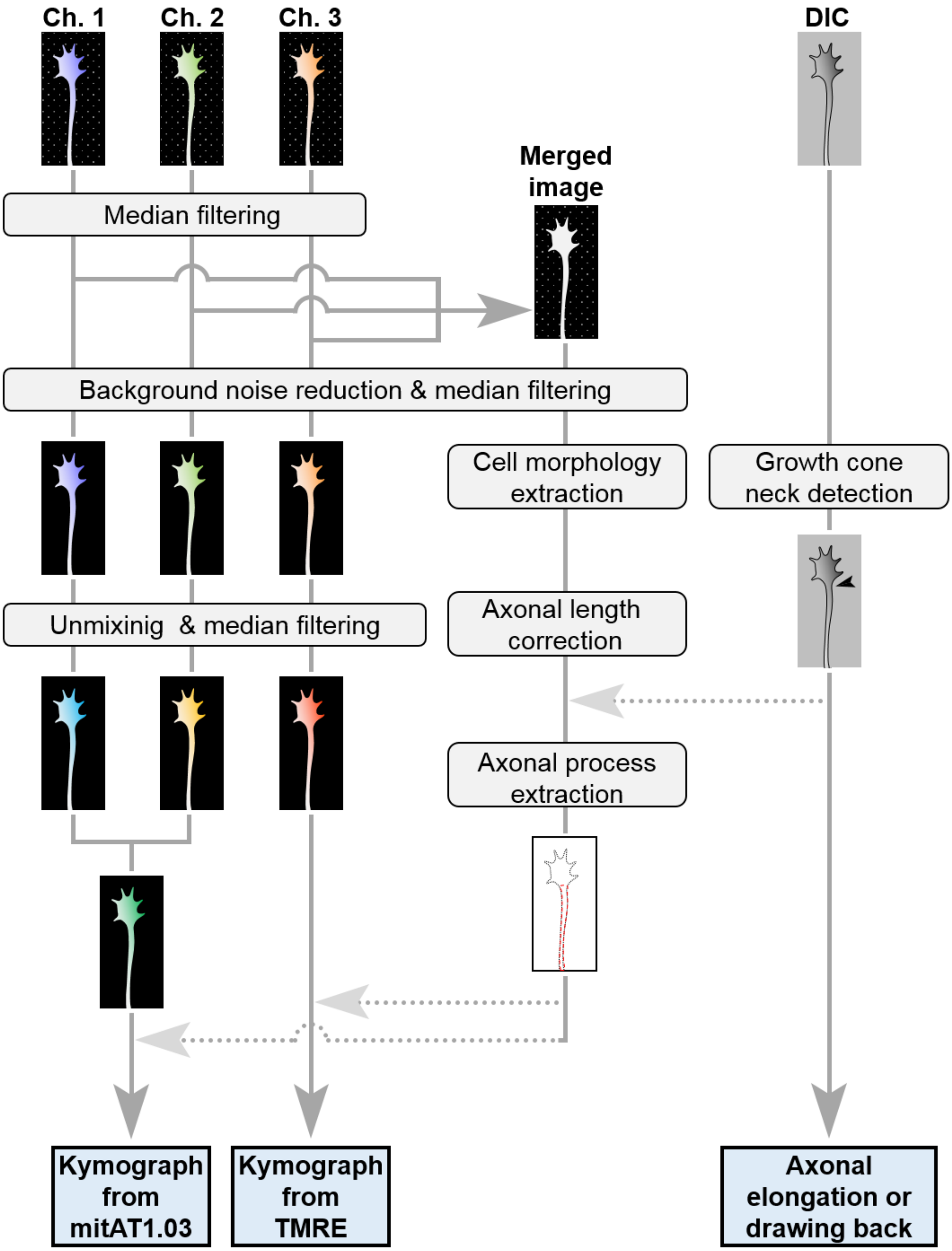

Supplementary Figure 14. Procedure of image processing.
Overview of our image processing software. Refer to Supplementary Figures S11, S12, and S13 for detail of fluorescent imaging and unmixing.
